# A perceptual map for gait symmetry quantification and pathology detection

**DOI:** 10.1186/s12938-015-0097-2

**Published:** 2015-10-29

**Authors:** Antoine Moevus, Max Mignotte, Jacques A. de Guise, Jean Meunier

**Affiliations:** Département d’Informatique & Recherche Opérationnelle (DIRO), Faculté des Arts et des Sciences, Université de Montréal, Montréal, QC H3C 3J7 Canada; Laboratoire de Recherche en Imagerie et Orthopédie, Centre de recherche du Centre Hospitalier de l’Université de Montréal (CRCHUM), Montréal, QC Canada

**Keywords:** Asymmetry map, Depth map, Gait analysis, Kinect depth sensor, Locomotor disorders, Multidimensional scaling (MDS), Perceptual color map, Temporal shift-invariance

## Abstract

**Background:**

The gait movement is an essential process of the human activity and the result of collaborative interactions between the neurological, articular and musculoskeletal systems, working efficiently together. This explains why gait analysis is important and increasingly used nowadays for the diagnosis of many different types (neurological, muscular, orthopedic, etc.) of diseases. This paper introduces a novel method to quickly visualize the different parts of the body related to an asymmetric movement in the human gait of a patient for daily clinical usage. The proposed gait analysis algorithm relies on the fact that the healthy walk has (temporally shift-invariant) symmetry properties in the coronal plane. The goal is to provide an inexpensive and easy-to-use method, exploiting an affordable consumer depth sensor, the Kinect, to measure the gait asymmetry and display results in a perceptual way.

**Method:**

We propose a multi-dimensional scaling mapping using a temporally shift invariant distance, allowing us to efficiently visualize (in terms of perceptual color difference) the asymmetric body parts of the gait cycle of a subject. We also propose an index computed from this map and which quantifies locally and globally the degree of asymmetry.

**Results:**

The proposed index is proved to be statistically significant and this new, inexpensive, marker-less, non-invasive, easy to set up, gait analysis system offers a readable and flexible tool for clinicians to analyze gait characteristics and to provide a fast diagnostic.

**Conclusion:**

This system, which estimates a perceptual color map providing a quick overview of asymmetry existing in the gait cycle of a subject, can be easily exploited for disease progression, recovery cues from post-operative surgery (e.g., to check the healing process or the effect of a treatment or a prosthesis) or might be used for other pathologies where gait asymmetry might be a symptom.

## Background

Scientists and medical communities have been interested in the analysis of gait movement for a long time, in particular because, as mentioned in [1–3] symmetrical gait is expected in the case of healthy people, whereas asymmetrical gait is a common feature of subjects with musculoskeletal disorders.

Abnormal or atypical gait can be caused by different factors, either orthopedic (hip injuries [4], bone malformations, etc.), muscular, or neurological (Parkinson’s disease, stroke [5], etc.). Consequently, different parts of the body can be involved or affected, which make gait analysis a complex procedure but also a reliable and accurate indicator for early detection (and follow-up) of a wide range of pathologies. It thus makes a 3D gait analysis (3DGA) procedure a powerful early clinical diagnostic tool [6] that is reliable and non-invasive, and which has been used successfully until now for screening test, detection and tracking of disease progression, joint deficiencies, pre-surgery planning, as well as recovery assessment from post-operative surgery or accident (rehabilitation). It is important to note that a gait analysis-based diagnostic tool also allows the reduction of the costs and amount of surgery per patient [7]. Also, a more appropriate medical prescription can be made by performing gait analysis before treating a patient, leading to a better recovery for the patients [6].

But nowadays, with the aging population, clinical diagnostics have to be cheaper, faster and more convenient for clinical [8–10] (or home [11]) usage while remaining accurate. However, analyzing a gait video sequence is often difficult, requires time, and subtle anomalies can be omitted by the human eye. Also, videos are not easy to annotate, store and share.

In this work, we focus on the design of both a reliable and accurate imaging system that is also inexpensive and easy to set up for daily clinical usage. This diagnostic tool is relying on the fact that the gait of healthy people is generally symmetrical in the coronal (frontal) plane (with half a period phase shift) and that asymmetrical gait may be a good indicator of pathologies and its progression [1–3, 5]. More precisely, the goal of our proposed GA-based diagnostic tool is to compute a perceptual color map of asymmetries from a video acquired by a depth sensor (Kinect) of a subject walking on a treadmill. The recording plane is the coronal plane in order to exploit the temporally shift-invariant properties of the movement. A perceptual color map of asymmetries is the compression of a subject’s video mapped into a single color image in such manner that asymmetries of the body movements in the human gait cycle may be clearly visible and immediately quantifiable.

This paper is organized as follows. First, “Previous work” section makes a study of existing procedures in the 3DGA literature. In “Proposed model” section, we introduce details about the dataset that will be used in our gait analysis system and we describe our asymmetry map estimation model based on the multidimensional scaling (MDS) mapping procedure and a local search refinement strategy. Finally, we show experimental results in “Results” section, give a discussion in “Discussion” section and conclude in “Conclusion” section.

### Previous work

Current 3DGA techniques can be divided in two categories, namely, with or without markers.

Among the state-of-the-art marker-based approaches, the Vicon motion-tracking and capture system [12] offers millimeter resolution of 3D spatial displacements. Due to its accuracy, it is often used as ground-truth for validation in medical application. On the other hand, the high cost of this system inhibits its widespread usage for routine clinical practices. Basically, optical motion capture system consists in tracking infrared (IR) reflective markers with multiple IR cameras [13]. Optical motion capture is efficient, but requires a lot of space, time, and expertise to be installed and used. For instance, placing the markers on the subject is prone to localization errors and requires someone who understands both the subject’s anatomy and the acquisition system. Also the subject might have to wear a special suit and change outfits, which is constraining both for the subject and for the physician.

Therefore, marker-less systems are a promising alternative for clinical environments and are often regarded as easy-to-set-up, easy-to-use, and non-invasive. They are either based on stereo-vision [14], structured light [15], or time-of-flight (TOF) [16] technologies. As a stereo-vision application, [17] used two camcorders to extract 3D information of the subjects and to measure the gait parameters. Although low-cost, the setup and calibration procedure of the system remains complex and only the lower parts of the body are measured. Also, stereo vision-based systems will not function properly if the subject’s outfit lacks texture. However, the Kinect sensor is based on structured light technology which makes it robust to textureless surfaces. The Kinect remains also compact and affordable. The Kinect has two output modes: depth map or skeleton modes. The former consists of an image sequence where the value of each pixel is proportional to the inverse of the depth, whereas the latter is a set of 3D points and edges that represents 20 joints of the human body.

Recent researches have been conducted to test whether the Kinect is suitable for clinical usage or not. Clark et al. [18] used the skeleton mode to measure spatial–temporal gait variability (such as the stride duration, speed, etc.) and compared it with data acquired by the high-end Vicon MX system. They found encouraging results for the estimation of the length of the steps and strides and the average gait velocity. Nevertheless, due to the inability of the skeleton tracking algorithm to accurately localize important anatomical landmarks on the foot, some spatio-temporal parameters of gait remain poorly estimated such as the assessment of step and the stride time. In addition, the Kinect camera was placed facing the subject, without a treadmill. Therefore the system was based on the analysis of only one gait cycle, because the intrinsic working range of the Kinect depth sensor is between 800 and 4000 mm. This somehow compromised the accuracy and the reliability of their system.

Gabel et al. [11] also used the skeleton mode to perform a 3DGA. They asked people to wear wireless sensors (gyroscopes and pressure sensors) at movement points and to walk back and forth along a straight path for approximately 7 min. They found that the Kinect was capable of providing accurate and robust results, but only a few gait parameters were tested and further research is under investigation. Finally, it is worth mentioning that none of the methods, using the Kinect skeleton mode, provide a visual feedback of the gait of the subject.

In [19], the authors compared the Kinect with depth map output mode versus a Vicon system. They placed two Kinects in a different alignment with the subject (facing and on the side) and measured key gait parameters, such as stride duration and length, and speed. They found excellent results with an average difference of less than 5 % for both Kinect camera setups. They also found that using the depth map data allows to reduce drastically the computation time for background removal.

In [10], the authors proposed to use a treadmill and a Kinect depth sensor to quantify the gait asymmetry with a low-cost gait analysis system. More precisely, the authors computed an index for quantifying possible asymmetries between the two legs by first dividing each gait cycle in two sub-cycles (left and right steps), and by comparing these two sub-cycles, in terms of an asymmetry index (proportional to the difference of depth, over a gait cycle, between the two legs) after a rough spatial and temporal registration procedure. Although the system is able to distinguish whether the subject has a symmetric walk or not, no visualization or information on the location of the asymmetries is provided, unlike our method.

In [9], the Kinect camera was placed at the back of a treadmill and used to record a video sequence of the subject’s walk. The authors then simply computed the mean of the obtained depth image sequence (over a gait cycle or a longer period) in order to compress the gait image sequence into one image which was finally called a depth energy image (DEI). Their results were conclusive since they were able to distinguish both visually and quantitatively asymmetries (a symmetric walk generating a DEI exhibiting a symmetric silhouette, in terms of mean depth and conversely). Nevertheless, this latter strategy is inherently inaccurate since taking the average (mean) depth over a gait cycle does not allow to detect all asymmetric body movements; indeed, movement variation of some parts of the body can clearly be different and asymmetric while keeping the same mean (in terms of mean depth).

**Fig. 1:**
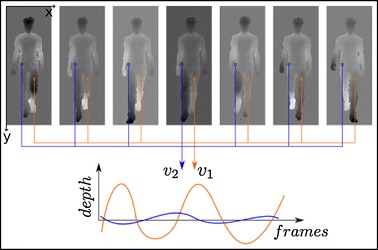
Example of two depth signals for a gait cycle of a subject

In our work, the depth image sequence of the gait, containing a certain number of gait cycles (wherein each pixel of the video corresponds to a depth signal as a function of time, as shown in Fig. 1) is reduced to three dimensions with a multi-dimensional scaling (MDS) mapping [20] using a temporally shift invariant Euclidean distance. This allows us to quickly display the gait image cube into an informative color image (with red, green and blue channels) allowing to visualize the asymmetric body parts of the gait cycle of a subject with a color difference, in a perceptual color space, which is linearly related to the asymmetry magnitude.

## Proposed model

### Data description

The dataset consists of multiple sequences of people walking on a treadmill, facing an inexpensive commercial depth sensor (Kinect). The Kinect sensor outputs 30 depth maps per second (30 *fps*), with a resolution of 640 per 480 pixels. The dataset contains 51 sequences acquired from 17 (healthy) subjects (17 males, 26.7 ± 3.8 years old, 179.1 ± 11.5 cm height and 75.5 ± 13.6 kg with no reported clinical asymmetry or gait impairment) walking with or without simulated length leg discrepancy (LLD). Every subject had to walk normally (group A), then with a 5 cm sole under the left foot (group B), then with the sole under the right foot (group C).

Sequences are approximately 5 min long and contain around 180 gait cycles. For all sequences, the same relative position between the treadmill and the sensor is kept in order for the subject to be within the same image area. The institutional ethical review board approved the study.

The method can be divided into four steps: a pre-processing for the silhouette extraction (“Silhouette extraction” section), a MDS-based mapping (“Multidimensional scaling-based mapping” section), a local search refinement strategy (“Refined estimation” section) and a color space conversion step (“Color space conversion” section).

### Silhouette extraction

Since the scene took place in a non-cluttered room where the treadmill is in the same position relatively to the camera, a 3D bounding box around the subjects can be set. Hence, by retrieving 3D information, we can convert this information back in the 2D image space to segment the subject’s silhouette directly from a depth map. To do so, the depth sensor is considered as a pinhole camera model with intrinsic parameters, *K*, (see [[21], p. 30]) defined as:

1 $$K = \left[ {\begin{array}{*{20}c} f & 0 & {c_{u} } \\ 0 & f & {c_{v} } \\ 0 & 0 & 1 \\ \end{array} } \right] = \left[ {\begin{array}{*{20}c} {575.82} & 0 & {240} \\ 0 & {575.82} & {240} \\ 0 & 0 & 1 \\ \end{array} } \right]$$

where *f* is the focal length in pixels and (*c*_*u*_, *c*_*v*_) is the image center in pixels (values given by the manufacturer). From a depth map, a pixel at position (*u*, *v*)^*T*^ with depth value, *d* is projected in 3D space, (*X*, *Y*, *Z*)^*T*^, using:

2 $$\left( {\begin{array}{*{20}c} X \\ Y \\ Z \\ \end{array} } \right) = dK^{ - 1} \left( {\begin{array}{*{20}c} u \\ v \\ 1 \\ \end{array} } \right) = d\left( {\begin{array}{*{20}c} {\frac{1}{f}*(u - c_{u} )} \\ {\frac{1}{f}*(v - c_{v} )} \\ 1 \\ \end{array} } \right)$$

First, the positions of the points around the subject, approximated by a 3D bounding box, are estimated. Second, the minimal and maximal depth, *Z*_*min*_ and *Z*_*max*_, of the eight points (of the bounding box) are retrieved. Third, the eight points were projected back in the 2D image space where the minimal and maximal vertical and horizontal 2D position value (*u*_*min*_, *u*_*max*_, *v*_*min*_, and *v*_*max*_) are finally estimated.

**Fig. 2:**
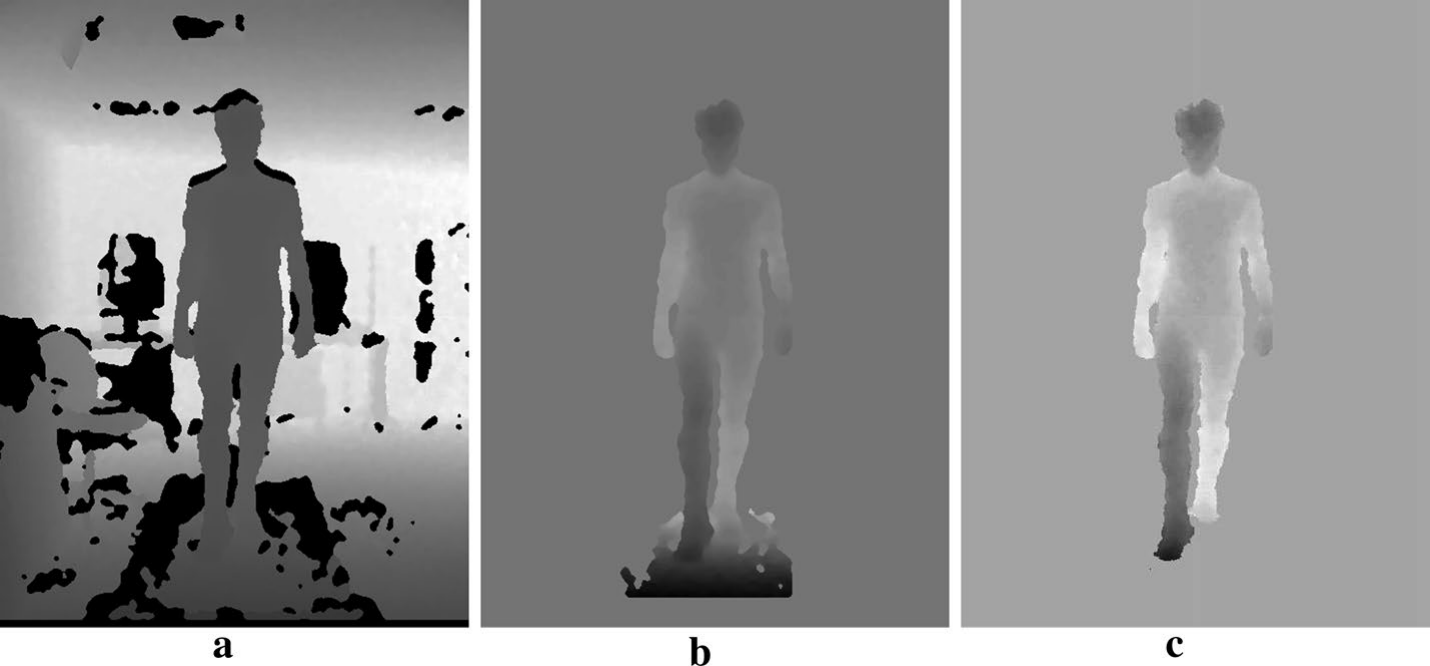
Setup and pre-processing steps. **a** Original depth map. **b** After clipping. **c** After treadmill removal

The necessity of working in 3D space is because of the spatial coherence of objects in the scene. For instance, in 3D space the treadmill is always beneath the subject whereas in an image it overlaps the subject, as shown in Fig. 2. Once this step is done, it is no more necessary to project the depth maps in the 3D space as long as the camera and the treadmill stay at the same relative position. In our case, some small adjustments of the enclosing parameters, *u*_*min*_, *u*_*max*_, *v*_*min*_, *v*_*max*_, *Z*_*min*_ and *Z*_*max*_, (bounding box) were needed to encompass all sequences.

Now, with, the required information, the subject can be segmented in each frame of the original gait depth sequence (of *N* frames).

#### Background removal

Background removal is trivial since the subject is in the middle of the image in a non-cluttered room. Therefore, every pixel outside the bounding box is clipped to a default value (see subsection “Clipping step” below).

#### Treadmill removal

After background removal, the only objects remaining in the image are the treadmill and the subject. Because the treadmill is below the subject, it can be removed by selecting pixels with coordinates superior to a threshold *T*_*y*_ (Y axis is going from top to bottom). An equation in the 2D-space can be derived from Eq. (2) in order to work directly on the image:

3 $$Y \, < \, T_{y}$$

4 $$\frac{d}{f}(v - c_v) < T_{y}$$

5 $$v \, < \frac{{fT_{y} }}{d} + c_v,\quad{\text{since}}\quad d > 0\;{\text{and }}f > 0$$

Figure 2 visually shows the different steps of the setup and pre-processing stage.

#### Clipping step

**Fig. 3:**
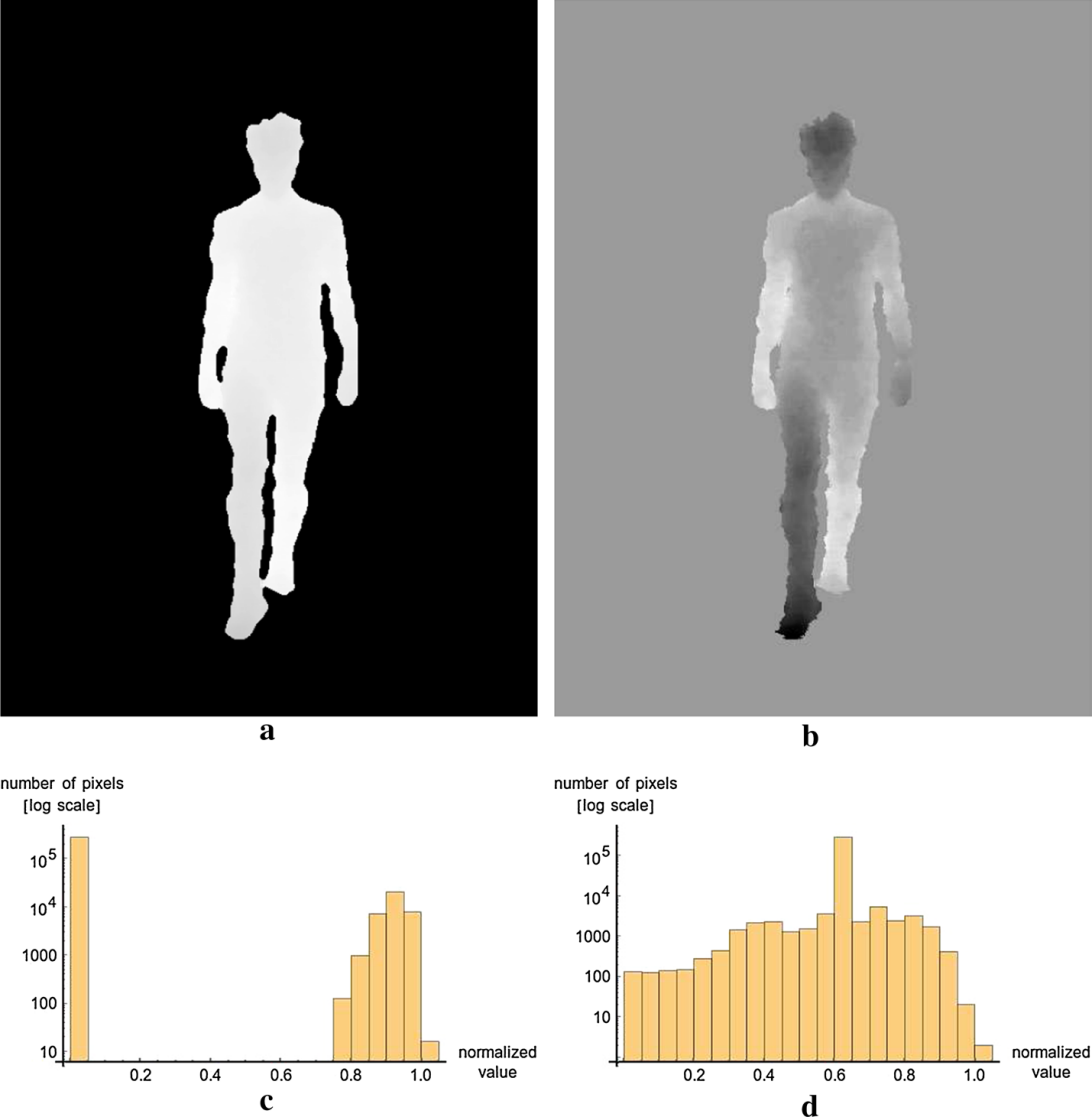
**a** An image where non-subject pixels where naively clipped to zero. The large difference of depth values, existing between the background and the subject, “squeezes” the depth values belonging to the subject and thus causes a decrease in depth resolution in this important and informative part of the image. **b** An image where background pixels where clipped to the mean (depth) value of subject’s pixels for the whole sequence. Spatial and depth resolution and image details are preserved. **c** The distribution of the pixel values of the image for the naive clipping (semi-log scale). **d** The distribution of the pixel values of the image for the smart clipping (semi-log scale). It is important to notice that the whole point of using a default clipping value is to make the distribution of pixel value of the images uni-modal and continuous. This will ensure a well contrasted map for the human eye

At this stage, it is important to recall that the core of the method, which is MDS- based, aims to preserve the pairwise distances between depth signals as well as possible in a final 3D (perceptual) color space. In order to get also an exploitable, high contrast (asymmetry) color image, that contains a wide dynamic range of color values (well distributed among all possible colors), the clipping value of the non- subject pixels have to be set carefully. Indeed, it is crucial that no big artificial pairwise distances are created, because those distances will induce a squeezing and penalizing effect on the other informative distances. For instance, if the relative value of the background differs a lot from the subject’s pixel (depth) values, the asymmetries between the right and left legs might not be easily distinguishable. Therefore the default background value has to be set carefully. In this work, this clipping value is estimated as being the mean of all the depth values belonging to the subject in the whole sequence (see Fig. 3).

#### Filtering step

Finally, the whole sequence is filtered with a 3D (3 × 3 × 3) median filter to remove some aberrations on the contours or on the top of the treadmill.

### Multidimensional scaling-based mapping

The MDS-based mapping technique [20] aims at visualizing the (temporally shift-invariant) motion asymmetric body parts with a perceptual color difference which corresponds (perceptually and linearly) to the asymmetry magnitude. This mapping is achieved by considering each pair of pixels (i.e., pair of *N*-dimensional depth signals) in the original gait video sequence and by quantifying their (temporally shift-invariant) degree of asymmetry with a temporally shift-invariant pairwise Euclidean distance $\beta_{{s_{ 1} ,s_{ 2} }}$ between each pair [**s**_1_(*t*), **s**_2_(*t*)] of depth signals:

6 $$\beta_{{s_{1} ,s_{2} }} = \mathop {\hbox{min} }\limits_{\tau } \left\{ {\left( {\mathop \sum \limits_{t = 0}^{N} {\mathbf{s}}_{1} (t + \tau ) - {\mathbf{s}}_{2} (t)} \right)^{2} } \right\}$$

where the maximal value of *τ* corresponds approximately to the number of frames in a gait cycle. In our application, *τ*_*max*_ = 66 and, in order to decrease the computational load, *τ* is increased with a step size equals to 6 (≈0.2 s).

In addition, four points are important to consider in this step:

- First, the use of the shift-invariant pairwise Euclidean distance is crucial in this MDS-based mapping step. Indeed, two pixels in the gait video cube, i.e., two depth signals (as a function of the time) with a perfect similar movement but in phase opposition (phase difference of half a gait cycle) like the legs and arms will have to be considered as symmetric with the same (perceptual) color in the final asymmetry map.
- Second, in order to provide a final perceptual color asymmetry visualization map, the MDS mapping is achieved in a perceptual color space, namely the classical CIE 1976 *L*∗, *a*∗, *b*∗ (LAB) color space which is approximately perceptually uniform. In this color space, a color difference shall (perceptually) appear twice as large for a measured (temporally shift-invariant) asymmetry value which is twice bigger.
- Third, as already said, MDS is a dimensionality reduction technique that maps objects lying in an original high *N* dimensional space to a lower dimensional space (3 in our application), but does so in an attempt that the between-signal distances are preserved as well as possible. The originally proposed MDS algorithm is not appropriate in our application and more generally for all large scale applications because it requires an entire *N* × *N* distance matrix to be stored in memory [with a *O*(*N*^3^) complexity]. Instead, we have herein adopted a fast alternative, FastMap [22], with a linear complexity *O*(*pN*) (with *p* = 3, the dimensionality of the target space). In FastMap, the axes of the target space are constructed dimension by dimension. More precisely, it implicitly assumes that the objects are points in a *p*-dimensional Euclidean space and selects a sequence of *p* ≤ *N* orthogonal axes defined by distant pairs of points (called pivots) and computes the projection of the points onto the orthogonal axes.
- The above-mentioned FastMap-based mapping method, which exploits an algebraic procedure, has the main advantage of being very fast (for large scale applications) but slightly less accurate than a MDS procedure exploiting a (gradient descent or a local search-based) optimization procedure [23]. For this reason, we decided to refine the estimated asymmetry map given by the FastMap as being the initial starting solution of a local search using a local exploration around the current solution. This step is now detailed in the following section.

### Refined estimation

#### Linear stretching

The FastMap-based mapping method allows us to preserve the between-depth-signal (shifted Euclidean) distances, as well as possible in a final 3D (perceptual LAB) color space with a scale factor *k*, which we have now to estimate in order to be able to refine the solution with a local search algorithm.

To this end, let **u** be the three-dimensional vector (**u** = (*L*, *A*, *B*)^*t*^) corresponding to the three *L*, *A*, *B* color bands of the final asymmetry image to be estimated and let also *β*_*s*,*t*_ denotes the Euclidean distance between two depth vectors, associated with a pair of sites at spatial (pixel) locations *s*, *t*. The scale factor *k* is the linear stretching factor which minimizes the following cost function:

7 $$\hat{k} = \mathop {{\text{argmin}}}\limits_{k} \underbrace {{\sum\limits_{{s,t_{{s \ne t}} }} {\left\{ {\overbrace {{k\beta _{{s,t}} }}^{{\beta _{{s,t}}^{{scaled}} }} - ||{\mathbf{u}}_{s} - {\mathbf{u}}_{t} ||_{2} } \right\}^{2} } }}_{{E_{o} }}$$

where the summation $\mathop \sum \nolimits_{{s,t_{s \ne t} }} {\text{is done}}$ over all pairs of sites existing in the final image to be estimated. In our application, in order to speed up this estimation procedure, we take the subset of pixel pairs induced by the graph presented in the following section. In this way, a simple local discrete grid search routine, for the parameter *k* in a suitable range (*k* ∈ [0 − 1] with a fixed step size set to 0.005) or a least square estimation can be easily achieved.

#### Local search refinement

**Fig. 4:**
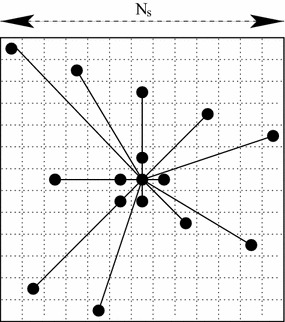
Spatial neighborhood used in our model. Each pixel is connected with its four nearest neighbors and *N*_*cnx*_ = 11 equally spaced other pixels located within a square neighbourhood window of fixed size *N*_*s*_ = 13 pixels

**Fig. 5:**
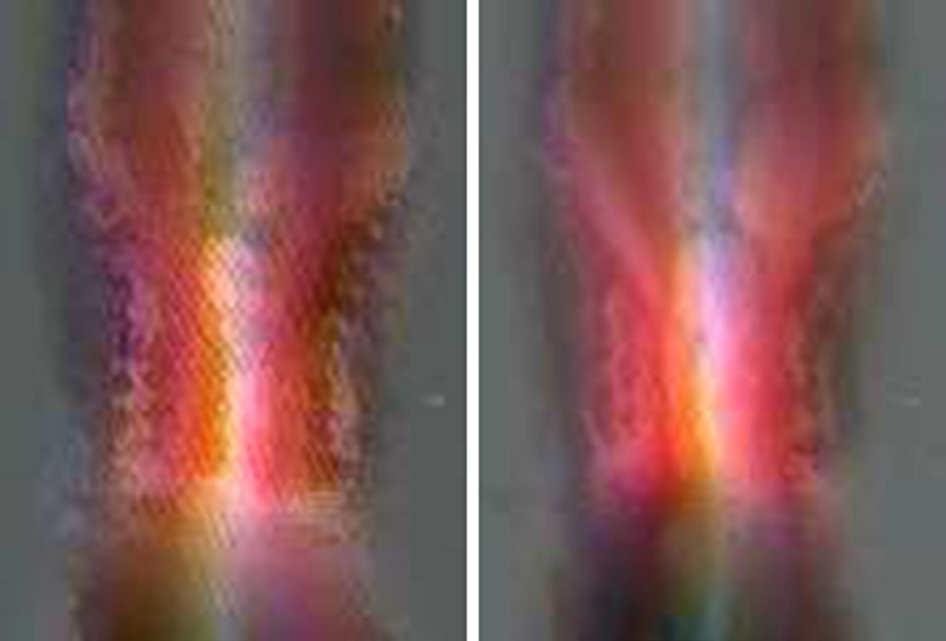
Magnified regions extracted from an asymmetry map (at lower leg level for the subject #S05 without LLD), obtained without and with (η = 0.025) a regularization term [see Eq. (9)]. The slight but annoying ringing noise effect has been corrected by the regularization term allowing us to a priori favor a edge-preserved asymmetry map that is piecewise smoothed

At this stage, we are very close to the solution of our optimization problem expressed in Eq. (7). To improve this solution, we use a deterministic local exploration around the current solution and a low radius of exploration (see the detailed Algorithm in “Appendix” and the validation of this local search procedure). For this local refinement, in order to decrease the computational load, we do not consider a complete graph but a graph in which each pixel is connected with its four nearest neighbors and *N*_cnx_ equally spaced other pixels located within a square neighborhood window of fixed size *N*_*s*_ pixels centered around the pixel (see Fig. 4). In addition, since this local search refinement strategy could be sensitive to noise, we add a regularization term allowing us to both incorporate knowledge concerning the types of estimated images a priori defined as acceptable solutions and to regularize the optimization problem. The regularization term used in our model is formulated in the (image) spatial domain and promotes a (regularized) estimated image **u** with spatial smoothness and edge-preserving properties (see Fig. 5). To this end, we have considered the generalized Gaussian Markov random field (GGMRF) regularization term initially proposed by Bouman and Sauer in tomographic reconstruction [24]:

8 $$\Omega \left( {\mathbf{u}} \right) = \mathop \sum \limits_{ < s,t > } \gamma_{st} \left| {{\mathbf{u}}_{s} - {\mathbf{u}}_{t} } \right|^{q}$$

where 1 ≤ *q* ≤ 2 is a parameter controlling the smoothness of the image to be estimated and/or the sharpness of the edges to be formed in the final estimated image. $\gamma_{st} = (2\sqrt 2 + 4)^{ - 1}\;{\text{or}}\;(4 + 4\sqrt 2 )^{ - 1}$ depends on whether the pair of neighboring sites (relative to the second order neighborhood system), or binary clique <*s*, *t*> is horizontal/vertical or right diagonal/left diagonal. This regularization term has the advantage of including a Gaussian MRF prior for *q* = 2 and a more interesting edge-preserving absolute-value potential function with *q* = 1 somewhat similar to the *L*_1_ regularizer proposed by Rudin et al. in [25]. In the regularization framework and under this constraint, an asymmetry map **u** can be seen as a solution to the following penalized cost function to be optimized:

9 $$\widehat{{\mathbf{u}}} = \mathop {{\text{argmin}}}\limits_{{\mathbf{u}}} \sum\limits_{{s,t_{s \ne t} }} {\left\{ {\beta_{s,t}^{scaled} - \| {\mathbf{u}}_{s} - {\mathbf{u}}_{{t}}\|_{2}} \right\}^{2} + \eta \mathop \sum \limits_{ < s,t > } \gamma_{st} \left| {{\mathbf{u}}_{s} - {\mathbf{u}}_{t} } \right|^{q} }$$

In this model, the set of *β*_*s*,*t*_, ({*β*_*s*,*t*_}) represents the *observed data*. The first term is related to the *preservation of between*-*depth signal distances* and can be viewed as a “goodness-of-fit” energy term. The second term corresponds to the regularization encoding some a priori expected properties of smoothness and of edge-preserving of the asymmetry image to be estimated. Let us also note that this model can be also easily viewed as a Bayesian optimization strategy formalizing a trade-off between a likelihood and an image prior expressing, via a prior distribution, that an acceptable estimated image is piecewise smoothed. *η* is the value controlling the contribution of these two terms.

### Color space conversion

It is important to mention that, at this stage, we are not assured that the LAB color values of the 3D asymmetry map are not saturated in the RGB space. In order to fix this problem, we use a simple linear stretching of the *L*, *A*, *B* color values such as *L* ∈ [0:100], and *A*, *B* have a maximal amplitude of 100 with a zero mean in order to ensure that a very small number of pixels are outside the RGB color space [23]. Once this linear stretching is achieved, a RGB conversion is done.

### Algorithm

Our model takes, on average, approximately 175 ± 10 s for a Core i7 Intel©, 4930 K CPU @ 3.40 GHz, 6803 bogomips and non-optimized code running on Linux. More precisely, the two steps; i.e., (1) estimations of the FastMap-based rough asymmetry map and (2) local search refining, takes, respectively, on average, 50 ± 5 and 125 ± 10 s for a 300 × 640 × 480 image sequence.

Let us add that the local search refining procedure can be easily computed in parallel. Indeed, the objective function to be minimized (Eq. 9) can be viewed as a Gibbs energy field related to a non-stationary Markov random field (MRF) model defined on a graph with long-range pairwise interactions (or binary cliques <*s*, *t*>). Each binary clique of this MRF model is associated to a non-stationary potential since this energy-based model is spatially variant and depends on the distance between the depth vectors associated with each pair of pixels *s*, *t*. Consequently, Algorithm 1 can be also viewed as a simple iterative conditional modes (ICM) procedure [26] for a MRF model with non-stationary and long-range pairwise interactions. Consequently, a Jacobi-type version of this Gauss–Seidel based ICM procedure (proposed in Algorithm 1) can be also efficiently implemented by using the parallel abilities of a graphic processor unit (GPU) (embedded on most graphics hardware nowadays available on the market) and can be greatly accelerated (up to a factor of 200) as proposed in [27].

Source code (in C++ language under Linux) of our algorithm with the set of image sequences are publicly available at the following http address: http://www.iro.umontreal.ca/∼mignotte/ResearchMaterial/pamga.html for the scientific community.

## Results

### Setup

This section presents the asymmetry maps obtained for the subjects with or without (simulated) pathologies. Sequences of 300 frames have been used (longer sequences did not yield significantly better results, see “Performance measures of the proposed model” section). This corresponds approximately to a range of 6–9 gait cycles depending on the subject’s speed and step size. On average for all images, the correlation score [23] (see end of “Local search algorithm” in “Appendix”) for the mapping of 300 frames to three color channels (according to our shifted Euclidean pairwise depth distance) is 93.5 ± 2 % which shows us that the FastMap-based MDS procedure is able to preserve a large quantity of information of the original image sequence (in terms of pairwise depth distances). We have used an offset of 400 frames (approximately 13 s) relatively to the beginning of the image sequence for all the subjects to allow them to get used to the treadmill. In addition, *η*, the value controlling the contribution between the likelihood and the regularization terms in Eq. (9) was set to *η* = 0.025 in all the following experiments.

### Initial tests

**Fig. 6:**
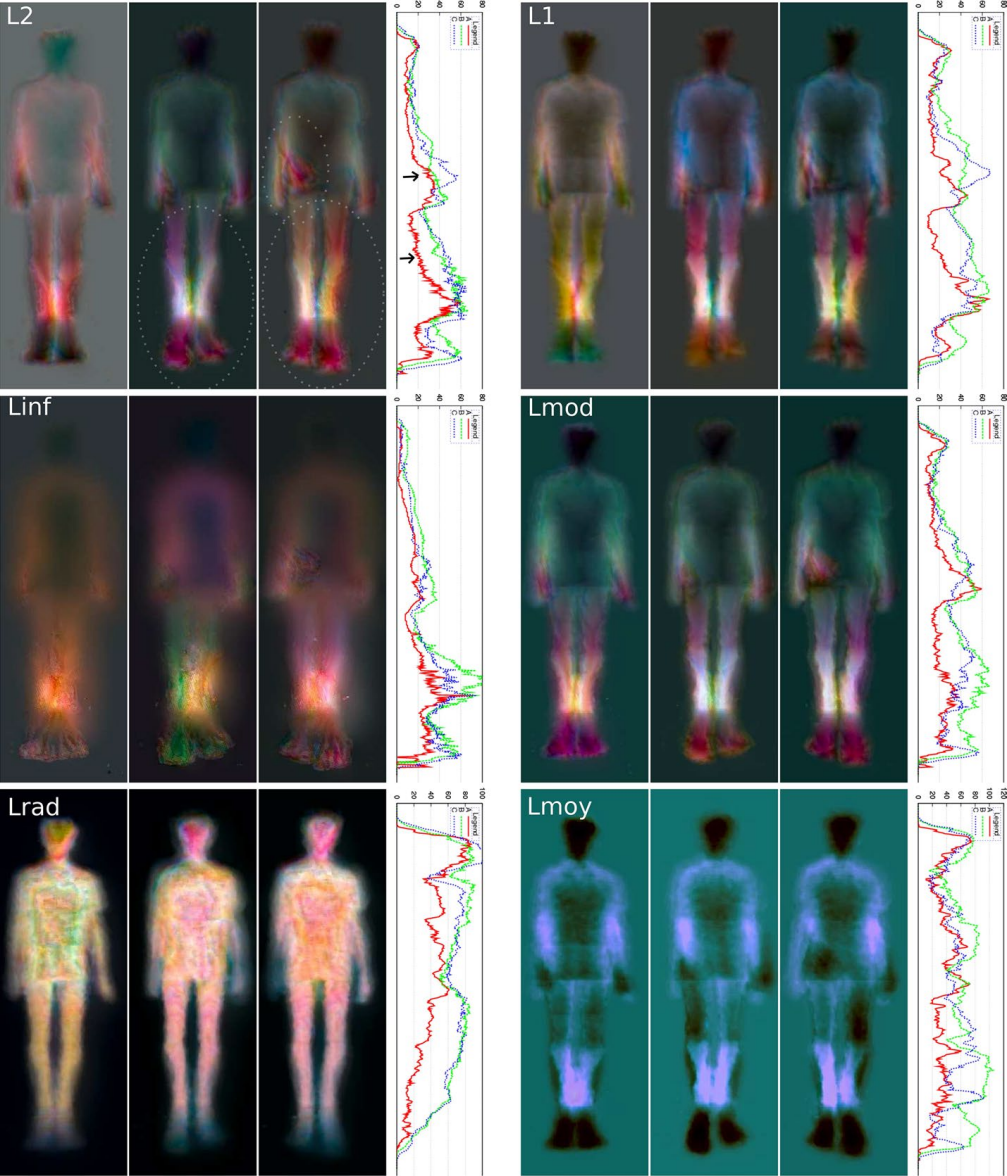
Asymmetry maps for subject #S05 for, respectively (from *left* to *right*) the normal gait and the* left* and* right *simulated LLD (cases A, B and C). The L2, L1, Linf, Lmod, Lrad and Lmoy distances are presented. For these cases, the ASI are, respectively 20.6/32.8/32.8, 22.3/35.6/30.9, 16.9/29.8/23.9, 20.9/30.3/29.2, 38.2/63.3/62.0 and 35.4/64.0/48.0. With* right* LLD (case C), the asymmetry of arm swing is clearly noticeable for the* right *LLD with the L2 (see the *circled* regions), L1 and Lmod distances. As expected, leg motion asymmetries are also visible for the *left* and *right* LLD. ASI curves are also presented (in which the *Y axis* shows the biggest mirrored difference (located on a *horizontal line*) as a function of the vertical distance from the top head of the subject (*X axis*) (see text)

In order to quantify the influence of the choice of the distance in the reliability of our asymmetry map, we have compared several (possibly shifted) distances. In addition to the shifted L2 norm (or Euclidean distance) between the two depth vectors, we have also considered; namely; the shifted L1 and L_inf_ (infinite) norms, the L2 norm between the amplitude of their Fourier spectrums (Lmod), which is inherently invariant to translation, the L2 norm between their amplitude histograms (Lrad) providing also a distance invariant to translation and finally the L1 norm between the mean of these two depth vectors (Lmoy). Figure 6 allows seeing the different asymmetry maps obtained for the subject #S05 of our database. Here the L2 distance shows clearly, as color differences between the left and right side of the body, the gait asymmetry magnitude. For instance, with right LLD (case C), the asymmetry of arm swing is clearly noticeable and for both right and left LLD (cases B and C) leg color differences (motion asymmetries) are visible.

Qualitatively, we can notice that the asymmetry maps based on the shifted L1 and Lmod distances visually appear as reliable as the asymmetry map given by the shifted L2 distance to detect motion asymmetries appearing as color differences between the left and right side of the body. In addition, we can also see quite clearly that the L_inf_ norm provides a (correlated) noisy asymmetry map (with artifacts and without edge preservation) with which we can however see color differences or the presence of asymmetry cues in the lower legs. The Lrad and Lmoy distances are clearly invariant to translation distances, nevertheless, the maps based on these two distances are inaccurate because they fail to detect all asymmetric body movements. Indeed, movement variation of some parts of the body can be different and asymmetric while keeping the same mean (depth) or keeping the same histogram. This explains why, with the right LLD (case C), the asymmetry of arm swing cannot be detected with the Lrad and Lmoy distances whereas this defect is easily detected and clearly visualized with the L2, L1 and Lmod based distances asymmetry maps.

### Performance measures of the proposed model

In order to get a quantitative measure of asymmetry, we propose to first estimate, for each line of the asymmetry map, the biggest mirrored differences. More precisely, for a line **k** of width *m*, (*m* being half the width of the asymmetry map) the set of biggest mirrored differences is:

$$\left\{ {{ \hbox{max} } ||p_{{i,{\mathbf{k}}}} - p_{{m - i,{\mathbf{k}}}} ||_{2} , \forall i \in [0,m/2]} \right\},$$

where *p*_*i*,*j*_ is a pixel value at position (*i*, *j*). This set of biggest mirrored differences yields a vertical curve whose mean amplitude, allows computing a global asymmetry index (ASI). From each asymmetry color map, this ASI curve is estimated by a two step procedure. Firstly, by estimating, individually for each subject, the position of the (symmetrical) longitudinal axis of his body (head to tail). This axis is determined from the silhouette contour (located in places of strong gradient) and its optimal position is searched on both sides (±10 pixels) around the vertical center line of the image (since this axis is assumed to be not too far from it) by estimating the vertical line whose pixel coordinates are the most symmetric with respect to the subject’s silhouette (body contour) in the median sense. Secondly, by seeking and recording the maximal color difference existing on either side (horizontally-oriented) of this preliminary estimated longitudinal axis. The ASI index is then the mean value of the ASI curve elements (see Algorithm “Estimation of the ASI” where the estimation procedure is outlined in pseudo-code).

**Fig. 7:**
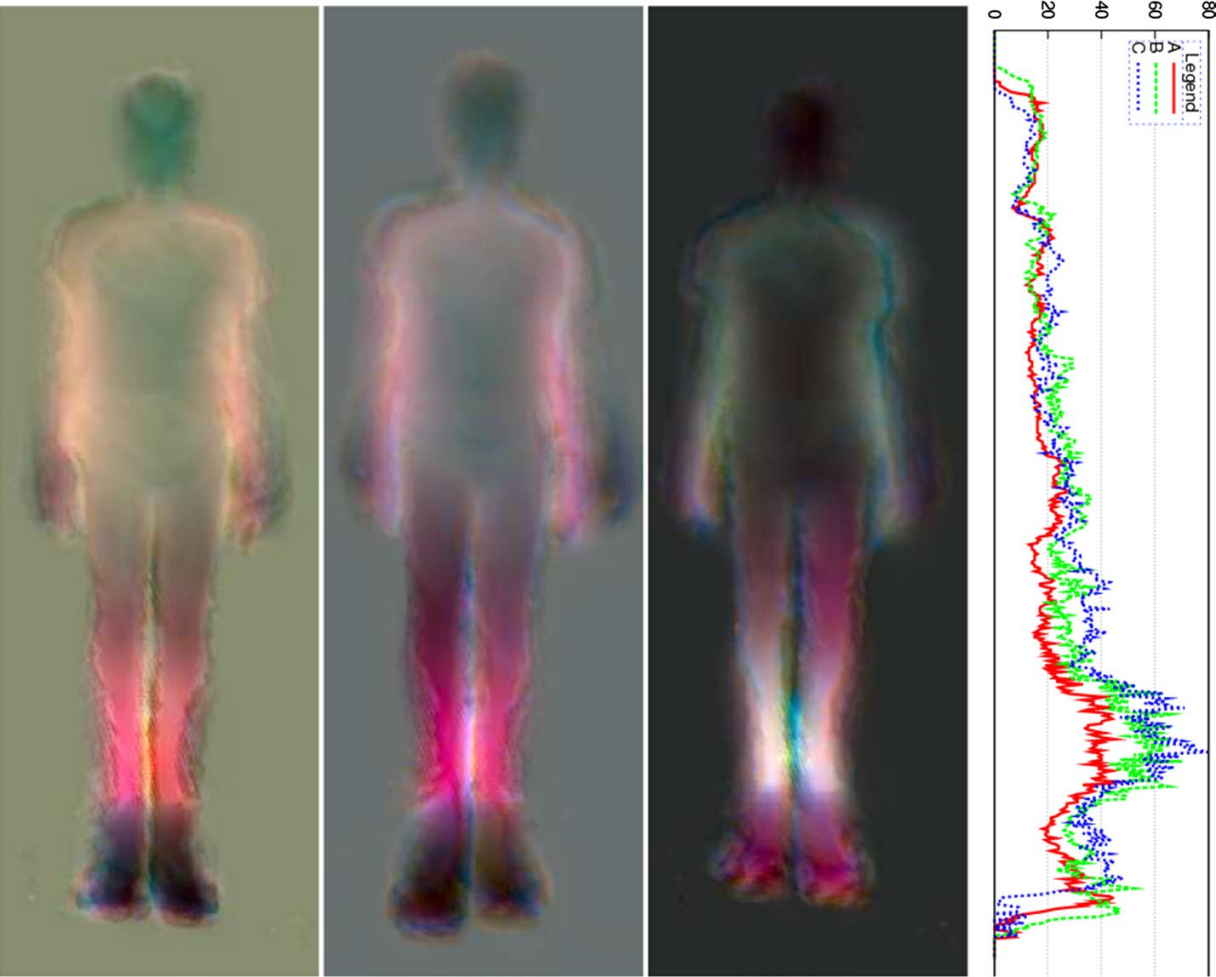
Asymmetry maps for subject #S02 for respectively (from *left* to *right*) the normal gait and with the *left* and *right* simulated LLD (cases A, B and C with L2 distance). For this case, the ASI is, respectively 21.2/27.8/28.5

**Fig. 8:**
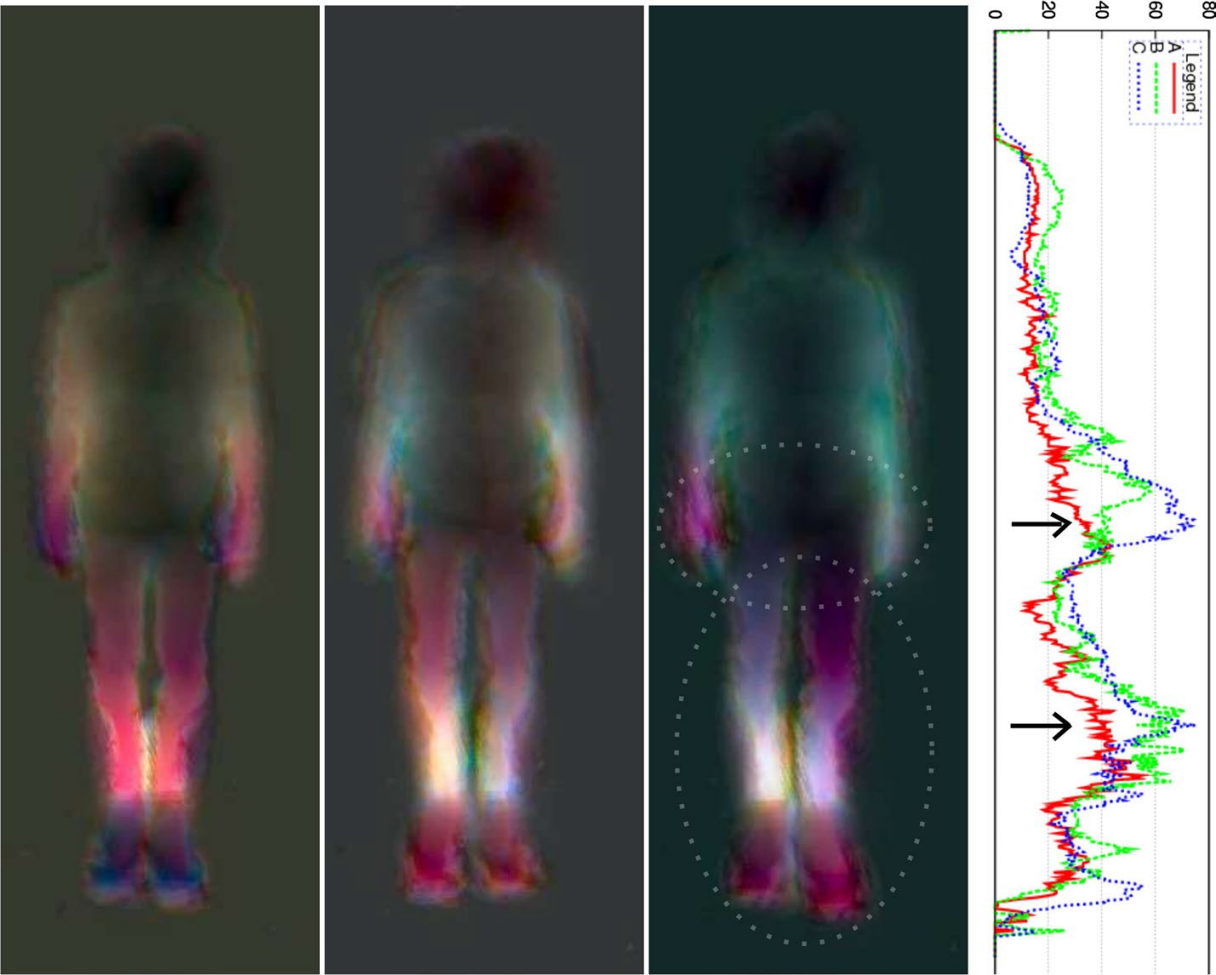
Asymmetry maps for subject #S12 for respectively (from *left* to *right*) the normal gait and with the *left* and *right* simulated LLD (cases A, B and C with L2 distance). For this case, the ASI is respectively 22.6/27.6/31.5

**Fig. 9:**
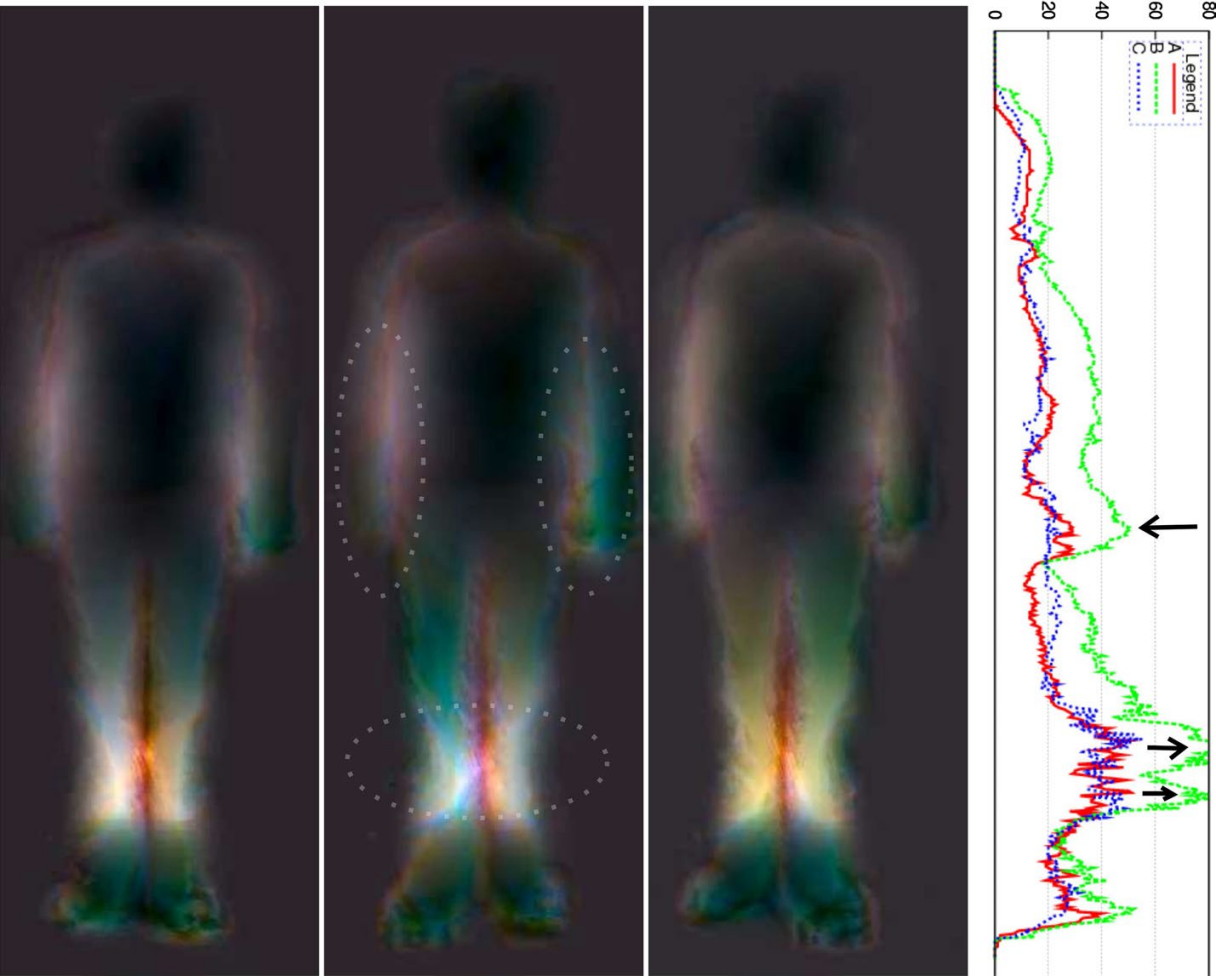
Asymmetry maps for subject #S11 for respectively (from *left* to *right*) the normal gait and with the *left* and *right* simulated LLD (cases A, B and C with L2 distance). For this case, the ASI is, respectively 19.9/35.1/20.0

**Fig. 10:**
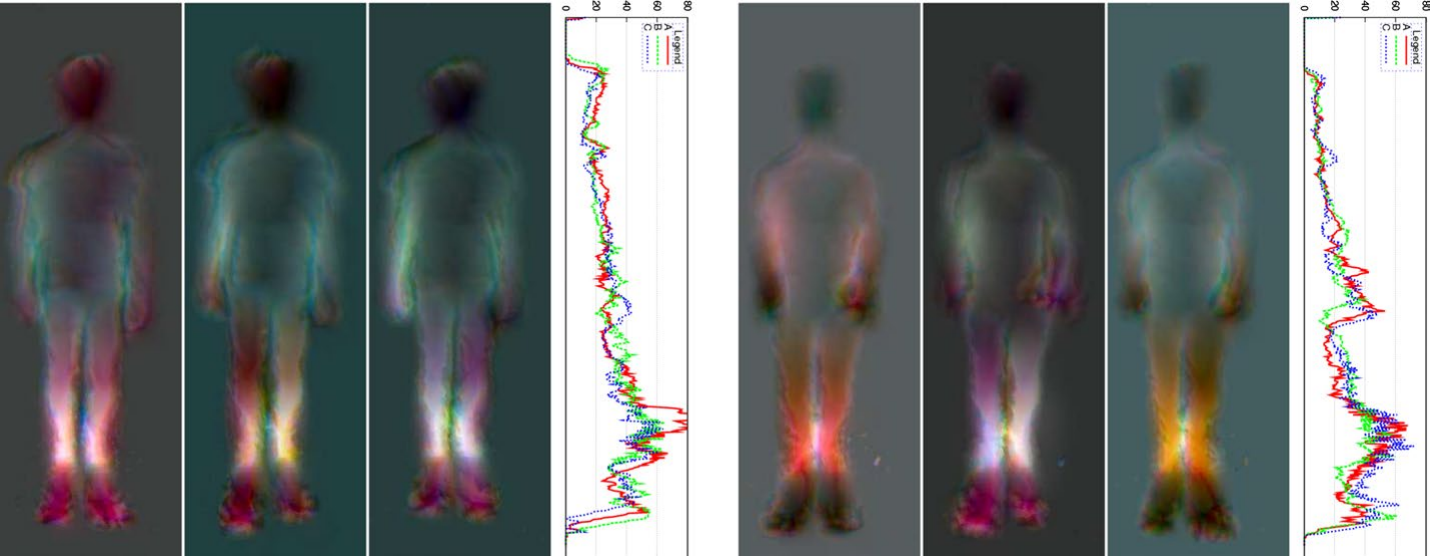
Asymmetry map for subjects #09 and #01, the two worst results of the dataset. The corresponding ASI is 29.04 for the normal gait, 30.37 for *left* LLD, and 25.98 for *right* LLD for subject #09. The subject had naturally a strong arm swing but a sole on the left foot seems to help rectifying it. Concerning the subject #01, the ASI is 24.22 for the normal gait, 21.60 for *left* LLD, and 23.96 for *right* LLD

**Fig. 11:**
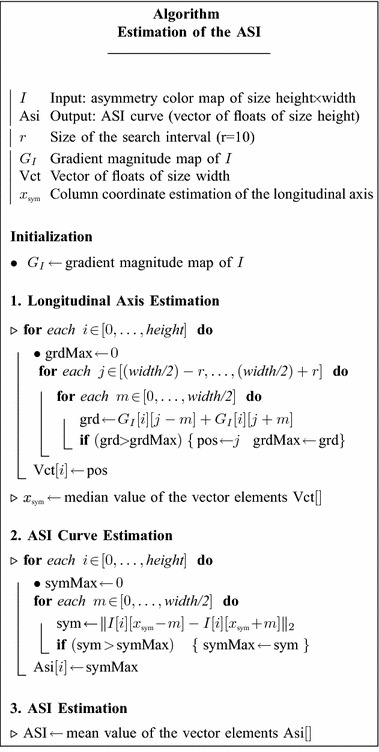
ASI curve and index estimation algorithm

**Fig. 12:**
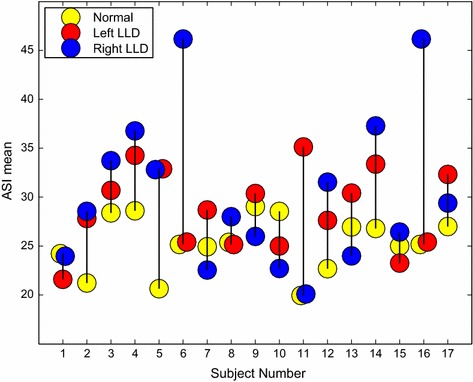
ASI index for the 17 subjects (normal gait and *left* and *right* LLD)

Asymmetries can be detected visually, as shown by Figs. 6, 7, 8, 9 and 10, but also quantitatively with the ASI curves [in which the Y axis shows the biggest mirrored difference, located on a horizontal line, as a function of the vertical distance from the top head of the subject (X axis)] or with the ASI index mentioned above (see Algorithm in Fig. 11 and the ASI index obtained by the 17 subjects of our experiment in Fig. 12). For instance, in Fig. 6, the ASI curves of the L2 distance display a significant gap between case A and cases B–C in the leg areas as expected (identified with an arrow in Fig. 6). Similarly, the asymmetry of arm swing appears as a gap between case A and C curves. In terms of paired differences t tests and confidence values, we can notice (see Table 1) that the shifted Euclidean distance seems to be the most appropriate distance allowing us to discriminate, on average with the ASI index, an asymmetry difference with a confidence value around 98.5−99 %. In this case, the refining step does not allow to (statistically significantly) increase or decrease this confidence score but this step actually allows us to include a regularization term in our energy-based model [see Eq. (9)] and to estimate a denoised and smooth asymmetry map with edge- preserving properties and consequently to somewhat remove the slight annoying ringing artifact effects occurring for some estimated asymmetry maps (see Fig. 5; Table 2).

**Table 1:** Critical values or cutoffs with the ASI index for paired t test

	L2	L1	L∞	LMod	LRad	LMoy
Before refining step						
Normal gait/left LLD	t = 2.763	t = 2.493	t = 0.781	t = 2.041	t = 1.311	t = 2.165
	98.6 %	97.6 %	54.4 %	94.2 %	79.2 %	95.4 %
Normal gait/right LLD	t = 3.225	t = 3.627	t = 2.058	t = 2.334	t = 1.650	t = 3.659
	99.5 %	99.8 %	94.4 %	96.7 %	88.2 %	99.8 %
After refining step						
Normal gait/left LLD	t = 2.906	t = 1.969	t = 2.260	t = 2.051	t = 0.7904	t = 2.296
	99.0 %	93.3 %	96.2 %	94.3 %	55.0 %	96.4 %
Normal gait/right LLD	t = 2.650	t = 2.868	t = 4.318	t = 2.985	t = 2.078	t = 4.155
	98.3 %	98.9 %	99.9 %	99.1 %	94.6 %	99.9 %

**Table 2:** Correlation *ρ* before the refining step and after the refining step for *η* = 0 and *η* = 0.025 for the first five subjects without the simulated length leg discrepancy (LLD)

	Before refining	η = 0	η = 0.025
Subject #01	ρ = 0.90	ρ = 0.91	ρ = 0.92
Subject #02	ρ = 0.89	ρ = 0.90	ρ = 0.90
Subject #03	ρ = 0.90	ρ = 0.91	ρ = 0.91
Subject #04	ρ = 0.86	ρ = 0.89	ρ = 0.88
Subject #05	ρ = 0.88	ρ = 0.89	ρ = 0.90
Average	ρ = 0.886	ρ = 0.900	ρ = 0.902

Table 3 shows the average (*µ*) and standard deviation (*σ*) of the ASI for the three groups of subjects (for the shifted *L*2 distance) and paired difference *t* test and confidence value for the paired tests *A* ≠ *B* and *A* ≠ *C*, respectively (1) without refining, (2) with *η* = 0 (with refining but without prior) and (3) *η* = 0.025 (with refining and prior). The statistical difference for the paired *t* test were highly significant (see also “T test table for 16 degree of freedom” in “Appendix”) for both left and right legs LLD groups (confidence value around 98.65 %). This demonstrates that this method can efficiently detect gait asymmetry. In practice, three subjects had a higher ASI for their normal gait than with LLD introduced with a sole (Fig. 10). By looking at their videos, the authors have noticed that those subjects already had a visible gait asymmetry (one arm swinging more than the other, tilted shoulders, etc).

**Table 3:** Statistics of the ASI index for the shifted L2 distance of 17 subjects

Without refining	Normal gait	Left LLD	Right LLD
Mean: µ	16.3135	19.0570	20.9700
Std. dev.: σ	2.4126	3.3141	6.7433
Cutoff		t = 2.763	t = 3.225
Confidence value		98.6 %	99.5 %

η = 0.0	Normal gait	Left LLD	Right LLD
Mean: µ	25.1076	28.9182	29.2358
Std. dev.: σ	3.0981	3.5568	6.0386
Cutoff :		t = 3.514	t = 2.526
Confidence value		99.71 %	97.75 %

η = 0.025	Normal gait	Left LLD	Right LLD
Mean: µ	25.2729	28.7888	30.3564
Std. dev.: σ	2.7439	3.942	7.5075
Cutoff :		t = 2.906	t = 2.650
Confidence value		99.0 %	98.3 %

We recall that we have used 300 frames in our application. For a sequence of 150 frames, the confidence value is 96.17 % and for a sequence of 600 frames, we obtained a confidence value of 99.52 but at the price of two times more computational load.

## Discussion

The preceding experimental results have shown that asymmetries can be detected visually with the proposed asymmetry maps, whether in terms of color differences with respect to the middle of the (standing) vertical axis but also with respect to the difference of length or geometric anatomical shapes or movements (legs and arms) exhibited on either side of the body (along this vertical axis), see for instance the difference of (1) length between the legs (for the L2 distance) in Fig. 6 with left and right LLD or for the subjects shown in Figs. 8, 9 and 10 or (2) length between the two arms in Fig. 7 or (3) mean gait posture (slightly inclined with respect to a vertical axis), for the subject shown in Fig. 10 (LLD only).

In addition, this asymmetry can be quantified with the proposed ASI index. It is also worth mentioning that the asymmetry map along with the ASI curve allows us to know where are distributed the asymmetric motions along the subject’s body. See for instance the circled areas and the gaps identified with arrows in these figures. The ASI curves thus provide quantitative local assessment of asymmetry. This cue could be a good indicator of pathologies and their progression over time for a more appropriate medical prescription leading to a better recovery for the subjects.

It is also important to understand that the VICON system is able to give very accurate but sparse (and generally not distributed equidistantly) measures (over the body) with which it is difficult to estimate a reliable dense asymmetry map without subsequent interpolation and extrapolation errors. By this fact, it makes a comparison between the Kinect and the VICON systems, in their ability to estimate an accurate gait asymmetric map, difficult to implement and to analyze. Although, it is also clear, that for a sufficient number of sensors distributed over the body, the VICON could be superior in terms of accuracy. Nevertheless, this late assertion does not detract from the originality of this work since the proposed estimation method of asymmetry map, based on the preservation of all the pairwise temporally shift invariant distances between depth signal as well as possible in a final 3D color space, with a MDS-based penalized likelihood strategy (and even the very concept of gait asymmetry map) has never been proposed to our knowledge, to date, and also remains inherently independent of the depth sensing technology used.

In our application, a paired sample *t* test is used to determine whether there is a statistically significant difference (increase) in the ASI index between the normal gait versus the left or right LLD (abnormal gait) groups and the p value (associated with this t test) actually quantifies the magnitude of this difference (i.e., a good confidence interval meaning that the difference is quite large). In our case, it just means that the increase in the ASI index, between the (*A* and *B*, *C*) groups, are statistically significant and then the asymmetry differences between these groups, in terms of ASI index, are real and are not due to standard error. Nevertheless, this does not mean that the ASI index can be used for separating normal from abnormal gait since, even if a majority of individuals have an ASI index above 30.00 for an abnormal gait (see Fig. 12 showing the scatter-plots of the ASI values for the different subjects with or without a LLD), there unfortunately are some subjects for which the normal gait remains more asymmetric (visually and in terms of ASI index) than some other subjects with a simulated LLD. In addition, as we have already mentioned in “Performance measures of the proposed model” section, that three subjects have a higher ASI for their normal gait than with LLD. More precisely, among the 17 subjects, three of them (#01, #09, #15) do not show a significant difference with or without LLD, two of them show a slight (but not significant) decrease in the ASI index with either the left or right simulated LLD (#07, #13) and one subject (#10), which has a visible gait asymmetry (one arm swinging more than the other along with tilted shoulders), has a higher ASI for his normal gait than with the right or left LLD introduced with a sole. Because of this fact, the ASI measure should not be used as an absolute measure for separating normal or abnormal gait, but rather as a *relative* measure, for example to analyze and quantify the gait recovery assessment through time or to check the adequacy of a prosthesis (or an adequate treatment) and to indicate, through an asymmetry map, where are located the strongest asymmetric areas of a subject’s gait cycle.

*η* remains the sole and major internal parameter of our model which acts as a regularization term and is fixed once and for all experimentation. Let us recall that the number of frames (*N* = 300) used in our MDS mapping should not be viewed as a critical internal parameter since doubling or halving the number of frames does not (significantly) change the efficiency of the FastMap mapping (see “Performance measures of the proposed model” section). Similarly, the two parameters of the spatial neighborhood (*N*_*s*_ = 13 and *N*_*cnx*_ = 11) used in our model are not sensitive parameters since the more connections we use, the better the convergence behavior of the algorithm is but at the cost of more computation time.

## Conclusion

In this paper, we have presented a new gait analysis system, based on Kinect depth sensor, which estimates a perceptual color map providing a quick overview of asymmetry existing in the gait cycle of a subject and an index (ASI), that was proved statistically significant with an approximately 98.75 % confidence value. While being inexpensive, marker-less, non-invasive, easy to set up and suitable for small room and fast diagnostic, this new gait analysis system offers a readable and flexible tool for clinicians to analyze gait characteristics which can be easily exploited for disease progression, recovery cues from post-operative surgery or might be used for other pathologies where gait asymmetry might be a symptom.

As future work, it would be necessary to validate the proposed method on real patients with different types of gait impairments. Besides, it would also be interesting to explore what could possibly be the other benefits of an asymmetry map estimation and visualization, which are not considered in this work, over a set of spatio-temporal gait parameters in a gait analysis system. An interesting research perspective would be specifically to analyze the topology and the pattern differences of these asymmetric areas in order to see if they are characteristic of a specific kind of disease (bone, neurodegenerative, muscular, etc.) or to simply determine, from the perceptual maps, that the asymmetry allows to localize the region of injury or to analyze the evolution of these asymmetric patterns through time to check the healing process or the effect of a treatment or a prosthesis.

## Figures and Tables

**Fig. 13:**
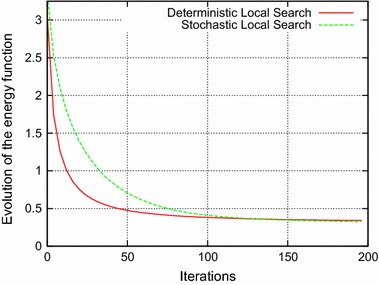
Evolution of the energy function expressed in Eq. (9) (for *η* = 0, r = 7, *N*_*cnx*_ = 11, *N*_*s*_ = 13) given by our deterministic local search procedure (see Algorithm 1) compared to a stochastic local search using the metropolis criterion (as proposed in [23]) with T_0_ = 1 and T_*f*_ = 10^−3^ (for the subject #05 without simulated length leg discrepancy)

**Fig. 14:**
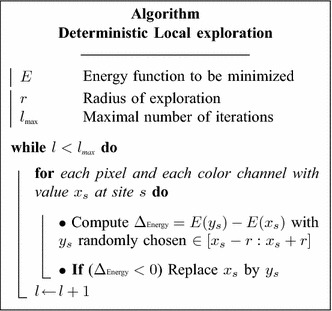
Local search algorithm

## Appendix

### Local search algorithm

There are two ways to validate the efficiency of our local search refinement strategy.

First in terms of the decrease of the cost function to be optimized [see Eq. (9)] with *η* = 0. To this end, Fig. 13 shows us the evolution of the energy function for our local search procedure (see Algorithm in Fig. 14) compared to a refinement strategy based on a (much more demanding) stochastic local search using the Metropolis criterion (as proposed in [23] or in [28]) with a starting temperature *T*_0_ = 1 (ensuring that, at the beginning of the stochastic search, approximately one-third of the sites change their luminance values between two complete image sweeps) and a final temperature *T*_*f*_ = 10^−3^ (ensuring that at the end of the stochastic search, very few sites change their channel color values). For this first experiment (and in all the following) experiments, we have considered a radius of exploration *r* = 7 and a graph in which each pixel is connected with its four nearest neighbors and *N*_cnx_ = 11 equally spaced other pixels located within a square neighborhood window of fixed size *N*_*s*_ = 13 pixels (see Fig. 4). In addition, we have considered a maximal number of iterations *l*_max_ = 200.

We can notice (see Fig. 14) that our proposed deterministic refinement strategy allows us to noticeably improve the solution of our energy-based estimation model compared to the solution given by the initial FastMap algorithm and to obtain, in our application, similar minimization results given by a stochastic approach (without the need to tune or fit parameters such as initial or final temperatures). This might be due to the (nearly) convex shape of our cost function around the solution given by the FastMap algorithm.

Another way to evaluate the efficiency of the rough initial FastMap mapping and then our proposed mapping refinement strategy consists in computing the correlation metric [29] which is simply the correlation of the temporally shift-invariant Euclidean distance between each pairwise depth vectors in the high (*N*-)dimensional space (let *X* be this vector) and their corresponding (pairwise) Euclidean distances in the low (3D) dimensional (LAB color) space (let *Y* be this vector). The correlation *ρ* can be estimated by the following equation:

10 $${{\rho}_{X,Y}} = corr(X,Y) = \frac{cov(X,Y)}{{\sigma_{X} \sigma_{Y} }} = \frac{{\frac{{X^{t} Y}}{\left| X \right|} - \overline{X}\;\overline{Y} }}{{\sigma_{X} \sigma_{Y} }}$$

where *X*^*t*^, |*X*|, $\overline{X}$ and *σX*, respectively represent the transpose, cardinality, mean, and standard deviation of *X*. This correlation factor (Pearson) will specifically quantify the degree of linear dependence between the variables *X* and *Y* and quantify how the FastMap technique is able to give a final 3D LAB mapping in which each pixel (LAB-)color value is set such that the between-color distances are preserved as well as possible [20]. A perfect correlation *ρ* = 1 indicates a perfect relationship between the initial set of between-depth signal distances and the final between-color distances in the final mapping (and a correlation of *ρ* = 0 indicates a total loss of information).

### Importing Kinect data

Each pixel of a depth image of the Kinect sensor is a 16-bit (little-endian) unsigned integer (uint16) (represented by four digits in hexadecimal base) which actually represents the depth in millimeter estimated at this location. To recover a binary image (of the sequence), three steps are thus required: the first one is to get to the starting byte of the image, the second one is to read and convert the pixels of the image, and the final one is to store each pixel value. To recover the *i*^*th*^ image, the starting point is evaluated as follows:

11 $${\text{image}}^i = 8+ ( 2 4+ 6 40 \times 4 80) \times i$$

For each pixel, four bytes are thus read sequentially and are converted into a decimal base according to the following scheme:

12 $$\begin{aligned} p_{{j_{read} }}^{i} &= \left[ {A123} \right]_{{{\text{little}} - {\text{endian}}}}^{\text{HEX}} \to 10 \times 16^{1} + 1 \times 16^{0} + 2 \times 16^{3} + 3 \times 16^{2} \hfill \\ &= [9121]^{\text{DECIMAL}} = p_{j}^{i} \hfill \\ \end{aligned}$$

where → designates the conversion of the read data to the decimal base. At this point the pixel $p_{{j_{{}} }}^{i}$ is stored as a unit16 in a table. This table represents the *i*^*th*^ image and once the *i*^*th*^ image is fully recovered, it is saved in a 3D array at the *i*^*th*^ position.

### T test table for 16 degree of freedom

Table 4 lists a few selected critical values (cutoffs) and the associated confidence values (in percentage) for a *t* distribution with 16 degrees of freedom (as in our case) for two-sided critical regions.

**Table 4:** Few selected critical values (cutoffs) and associated confidence values (in percentage) for a two-tailed test and for a t-distribution with 16 degrees of freedom

80 %	90 %	95 %	98 %	99 %	99.5 %	99.8 %	99.9 %
1.337	1.746	2.120	2.583	2.921	3.252	3.686	4.015

In order to estimate the confidence values between two known values of cutoffs we have used, in our application, a Lagrange method based interpolation leading to the following polynomial interpolation:

13 $$\begin{aligned} &- 1 5. 7 3 6 3+ 1 1 9. 50 2x - 3 8. 2 8 7 5x^{ 2} - 2. 6 8 9 1 6x^{ 3} + 4. 8 8 6 9 5x^{ 4} - 1. 3 1 2x^{ 5} \hfill \\ & \quad + 0. 1 5 2 7 5x^{ 6} - 0.00 6 7 6 90 3x^{ 7} \hfill \\ \end{aligned}$$

## References

[1] Engsberg JR, Tedford KG, Harder JA, Mills JP. Timing changes for stance, swing, and double support in a recent below-knee-amputee child. Pediatr Exerc Sci. 1990;2(3):255–62. 10.1123/pes.2.3.255 39152591

[2] Loizeau J, Allard P, Duhaime M, Landjerit B (1995). Bilateral gait patterns in subjects fitted with a total hip prosthesis. Arch Phys Med Rehabil.

[3] Hamill J, Bates B, Knutzen K (1984). Ground reaction force symmetry during walking and running. Res Q Exerc Sport.

[4] Miki H, Sugano N, Hagio K, Nishii T, Kawakami H, Kakimoto A, Nakamura N, Yoshikawa H (2004). Recovery of walking speed and symmetrical movement of the pelvis and lower extremity joints after unilateral THA. J Biomech.

[5] Alexander LD, Black SE, Patterson KK, Gao F, Danells CJ, McIlroy WE (2009). Association between gait asymmetry and brain lesion location in stroke patients. Stroke.

[6] Wren TA, Gorton GE, Ounpuu S, Tucker CA (2011). Efficacy of clinical gait analysis: a systematic review. Gait Posture.

[7] Wren TA, Kalisvaart MM, Ghatan CE, Rethlefsen SA, Hara R, Sheng M, Chan LS, Kay RM (2009). Effects of preoperative gait analysis on costs and amount of surgery. J Pediatr Orthop..

[8] Carse B, Meadows B, Bowers R, Rowe P (2013). Affordable clinical gait analysis: an assessment of the marker tracking accuracy of a new low-cost optical 3d motion analysis system. Physiotherapy..

[9] Rougier C, Auvinet E, Meunier J, Mignotte M, de Guise JA. Depth energy image for gait symmetry quantification. In: Engineering in Medicine and Biology Society, EMBC, 2011 annual international conference of the IEEE. IEEE; 2011, p. 5136–9. 10.1109/IEMBS.2011.6091272 22255495

[10] Auvinet E, Multon F, Meunier J. Lower limb movement asymmetry measurement with a depth camera. In: Engineering in Medicine and Biology Society (EMBC), 2012 annual international conference of the IEEE; Aug 2012, p. 6793–6. 10.1109/EMBC.2012.6347554 23367489

[11] Gabel M, Gilad-Bachrach R, Renshaw E, Schuster A. Full body gait analysis with kinect. In: Engineering in Medicine and Biology Society (EMBC), 2012 Annual International Conference of the IEEE. IEEE; 2012, p. 1964–7. 10.1109/EMBC.2012.6346340 23366301

[12] Motion capture systems from vicon. http://www.vicon.com/. Accessed 26 Oct 2015.

[13] Potdevin F, Gillet C, Barbier F, Coello Y, Moretto P. The study of asymmetry in able-bodied gait with the concept of propulsion and brake. 9th symposium on 3D analysis of human movement, Valenciennes, France; 2006.

[14] Lazaros N, Sirakoulis GC, Gasteratos A (2008). Review of stereo vision algorithms: from software to hardware. Int J Optomechatr.

[15] Salvi J, Pages J, Batlle J (2004). Pattern codification strategies in structured light systems. Pattern Recogn.

[16] Hansard M, Lee S, Choi O, Horaud R (2013). Time-of-flight cameras.

[17] Leu A, Ristic-Durrant D, Graser A. A robust markerless vision-based human gait analysis system. In: 2011 6th IEEE international symposium on applied computational intelligence and informatics (SACI), May 2011, p. 415–20.

[18] Clark RA, Bower KJ, Mentiplay BF, Paterson K, Pua Y-H (2013). Concurrent validity of the microsoft kinect for assessment of spatiotemporal gait variables. J Biomech.

[19] Stone EE, Skubic M. Evaluation of an inexpensive depth camera for passive in-home fall risk assessment. In: Pervasive Health; 2011, p. 71–7.

[20] Cox TF, Cox MA (2000). Multidimensional scaling.

[21] Ponce J, Forsyth D (2003). Computer vision: a modern approach.

[22] Faloutsos C, Lin K-I. FastMap: a fast algorithm for indexing, data-mining and visualization of traditional and multimedia datasets. ACM; 1995, vol 24, no 2, p. 163–74.

[23] Mignotte M (2012). A bicriteria-optimization-approach-based dimensionality-reduction model for the color display of hyperspectral images. IEEE Trans Geosci Remote Sensing.

[24] Bouman CA, Sauer K (1996). A unified approach to statistical tomography using coordinate descent optimization. IEEE Trans Image Process.

[25] Rudin L, Osher S, Fatemi E (1992). Nonlinear total variation based noise removal algorithms. Phys D.

[26] Besag J (1986). On the statistical analysis of dirty pictures. J R Stat Soc.

[27] Jodoin P-M, Mignotte M (2006). Markovian segmentation and parameter estimation on graphics hardware. J Electr Imaging..

[28] Moevus A, Mignotte M, de Guise J, Meunier J. Evaluating perceptual maps of asymmetries for gait symmetry quantification and pathology detection. In: 36th international conference of the IEEE engineering in medicine and biology society, EMBC’2014, Chicago, August 2014. 10.1109/EMBC.2014.6944332 25570700

[29] Jacobson NP, Gupta MR (2005). Design goals and solutions for display of hyperspectral images. IEEE Trans Geosci Remote Sensing.

